# The Role of Sclerostin in Bone and Ectopic Calcification

**DOI:** 10.3390/ijms21093199

**Published:** 2020-04-30

**Authors:** Annelies De Maré, Patrick C. D’Haese, Anja Verhulst

**Affiliations:** Lab of Pathophysiology, Faculty of Pharmaceutical, Biomedical and Veterinary Sciences, Campus Drie Eiken, University of Antwerp, 2610 Antwerp, Belgium; annelies.demare@uantwerpen.be (A.D.M.); patrick.dhaese@uantwerpen.be (P.C.D.)

**Keywords:** sclerostin, vascular calcification, Wnt signaling

## Abstract

Sclerostin, a 22-kDa glycoprotein that is mainly secreted by the osteocytes, is a soluble inhibitor of canonical Wnt signaling. Therefore, when present at increased concentrations, it leads to an increased bone resorption and decreased bone formation. Serum sclerostin levels are known to be increased in the elderly and in patients with chronic kidney disease. In these patient populations, there is a high incidence of ectopic cardiovascular calcification. These calcifications are strongly associated with cardiovascular morbidity and mortality. Although data are still controversial, it is likely that there is a link between ectopic calcification and serum sclerostin levels. The main question, however, remains whether sclerostin exerts either a protective or deleterious role in the ectopic calcification process.

## 1. Discovery of Sclerostin

In the 1950s, sclerosteosis and van Buchem disease, two rare autosomal recessive disorders characterized by massive and progressive bone overgrowth, were described for the first time [1,2]. Since both diseases are clinically and radiographically very similar, it was speculated that these two conditions result from mutations in the same gene [3]. Genome-wide linkage analysis on both sclerosteosis and van Buchem disease patients indeed revealed co-localization of the disease gene loci in the chromosomal region 17q12-q21 [4,5]. In sclerosteosis patients, extensive sequence analysis in this chromosomal region identified loss-of-function mutations in a previously unknown gene, now called the *SOST* gene [6]. In van Buchem disease patients, however, no disease-causing mutations were found within this gene [6,7]. By further sequencing downstream of the *SOST* gene, a 52kb deletion (containing a regulatory element) was identified, which affects the transcription of the *SOST* gene in bone [8,9,10].

The *SOST* gene product ‘sclerostin’ is a 22-kDa protein, and is a well-known negative regulator of bone formation. Although generally viewed as an osteocyte-specific protein, other tissues such as the kidney, liver, bone marrow, lung, heart and pancreas also express *SOST* mRNA [6,7]. In contrast to sclerosteosis patients, in which functional sclerostin is completely absent, van Buchem disease patients have a reduced sclerostin expression compared to healthy controls [6,11]. This is in line with the milder clinical phenotype that is observed in van Buchem disease patients, compared to sclerosteosis patients.

## 2. The Role of Sclerostin in Physiological Calcification

The canonical Wnt/β-catenin signaling pathway, in addition to its function during embryogenesis [12,13], also plays a crucial role in adult tissue homeostasis by regulating the maintenance and differentiation of stem cells. In particular, this signaling cascade also exerts an important regulatory pathway in the differentiation of mesenchymal stem cells towards the osteoblast-lineage.

Beta-catenin is the central regulatory player in the canonical Wnt signaling. Activation of this signaling cascade, by binding of the Wnt ligands to the Frizzled (Fz) receptor and Low-density Lipoprotein Receptor-related Protein 5/6 (LRP5/6) co-receptors, leads to inhibition of the β-catenin degradation complex. In this way, β-catenin can accumulate in the cytoplasm, and subsequently be translocated into the nucleus. In the nucleus, β-catenin functions as a coactivator of the transcription factors T-cell factor (TCF) and Lymphoid Enhancer-binding factor (LEF), thereby modifying gene transcription. It has been shown that the Wnt/β-catenin signaling cascade downregulates adipogenic differentiation by inhibiting the expression of Peroxisome Proliferator-Activated Receptor gamma (PPARγ) and CCAAT/Enhancer Binding Protein alpha (C/EBPα), both important adipogenic regulators, while stimulating Runt-related transcription factor 2 (Runx2) and Osterix, well-known inducers of osteogenesis [14,15]. The canonical Wnt signaling also stimulates osteoblast maturation and viability of osteoblasts and osteocytes. These cells then increase their production of osteoprotegerin (OPG) (a decoy receptor of Receptor Activator of Nuclear Factor Kappa-Β Ligand (RANKL)), by which osteoclast formation is inhibited.

**Figure 1:**
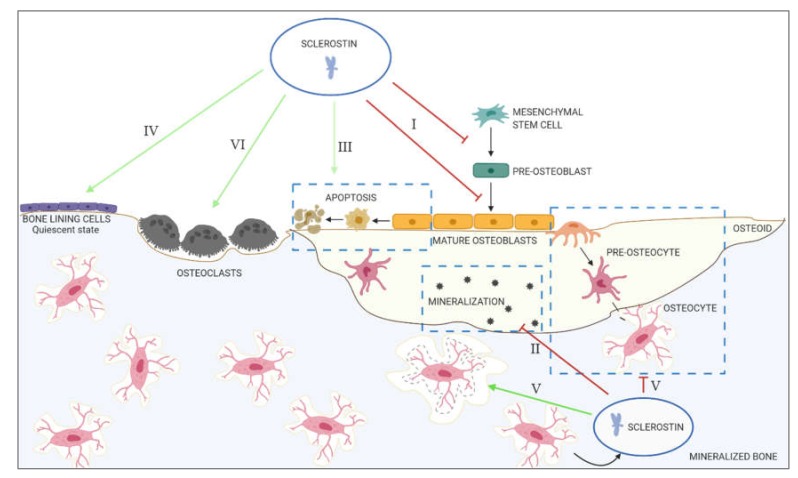
Overview of the actions of sclerostin in the bone. I: Inhibition of proliferation and differentiation of osteoprogenitor/pre-osteoblastic cells, as well as decreased activation of mature osteoblasts; II: decreased mineralization; III: increased apoptosis of the osteogenic cells; IV: maintenance of bone lining cells in their quiescent state; V: regulation of osteocyte maturation and osteocytic osteolysis; VI: stimulation of bone resorption.

To prevent excessive bone formation, several antagonists are produced amongst which is sclerostin. Mechanical unloading [16], low levels of serum parathyroid hormone (PTH) [17,18] and estrogen deficiency [19] trigger sclerostin production. As already mentioned above, in the bone, sclerostin is mainly produced by the osteocytes, the cells that reside within the bone matrix and comprise between 90%–95% of all bone cells. After its secretion, sclerostin will be anchored to the LRP4 receptor on the osteoblast membrane, by which sclerostin is retained in the bone compartment [20]. Sclerostin can also bind to LRP5/6, leading to receptor internalization and/or reduced availability of these co-receptors to Wnt ligands, which results in inhibition of the canonical Wnt signaling. This leads to (Figure 1):

 **I.**  **Inhibition of proliferation and differentiation of osteoprogenitor/pre-osteoblastic cells, as well as decreased activation of mature osteoblasts**

Osteoblasts are derived from mesenchymal stems cells, which are multipotent progenitor cells that are able to differentiate into a variety of cell types (including osteoblasts, chondrocytes, adipocytes, smooth muscle cells [21] and endothelial cells [22]). Depending on the specific activation of signaling pathways (such as Wnt/β-catenin signaling) and transcription factors (such as Runx2 and osterix), mesenchymal cells will commit to the osteoblastic lineage. Inhibition of the canonical Wnt signaling by sclerostin therefore directly prevents the development of new osteoblasts. However, Thouverey and Caverzasio found that sclerostin not only functions by inhibiting canonical Wnt signaling, but also activates platelet-derived growth factor receptor signaling to inhibit osteoblast differentiation [23]. Sclerostin also inhibits the activity of mature osteoblasts, since osteocalcin, procollagen type 1 N-terminal Propeptide (P1NP) and bone-specific alkaline phosphatase (BsAP), all produced by the osteoblast and therefore considered indicators of osteoblastic activity, were increased after administration of romosozumab, an antibody directed against sclerostin [24].

 **II.**  **Decreased mineralization**

Mineralization of newly formed bone is a dynamic process in which Small Integrin-Binding Ligand N-linked Glycoproteins (SIBLINGS), such as Matrix Extracellular Phosphoglycoprotein (MEPE), are involved [25]. A key characteristic of MEPE, and several other SIBLING proteins, is the presence of an Acidic Serine Aspartate-Rich MEPE-associated (ASARM) motif [26]. When cleaved by cathepsin B, the ASARM motif inhibits mineralization and phosphate uptake [27]. Cleavage of this ASARM motif can be prevented by Phosphate-regulating neutral Endopeptidase (PHEX), which binds to full-length MEPE [28,29]. Regulation of mineralization is therefore determined by the PHEX/MEPE ratio. Sclerostin is involved in the regulation of this PHEX/MEPE axis, by inducing the expression of ASARM-peptides (anti-mineralization) and downregulating PHEX (pro-mineralization) [30,31].

 **III.**  **Increased apoptosis of the osteogenic cells**

Experiments in *Sost* knockout (KO) mice demonstrated decreased apoptosis of osteoblasts and osteocytes, while osteoblast activity was increased in these animals compared to wild type (WT) mice [32]. These results were confirmed by Chandra et al., showing that in a mouse model of radiation damage, the inhibition of sclerostin protected against apoptosis of osteoblasts [33]. Additionally, in human osteoblastic cells, sclerostin induced apoptosis by activating caspases 1, 3, 4 and 7, as well increasing the expression of the pro-apoptotic factor Bax [34].

 **IV.**  **Maintenance of bone lining cells in the quiescent state**

Bone lining cells are found covering the bone surface. They are considered to be derived from previously active osteoblasts that did not undergo apoptosis or differentiated into osteocytes [35]. During normal physiology, active bone lining cells play a crucial role as coordinators of bone formation and resorption [36,37]. Regulation of the activity state of the bone lining cells—either remaining in the quiescent state or being reactivated to osteoblasts—at least in part, seems to be controlled by sclerostin. One study, performed in ovariectomized rats and cynomolgus monkeys, demonstrated that anti-sclerostin antibody treatment strongly reduced quiescent bone surfaces, whilst increasing bone surfaces actively involved in mineralization [38]. Another study in which mice were administered with an anti-sclerostin antibody, showed reactivation of the bone lining cells, as indicated by the increase in size and expression of osteocalcin, a marker of osteoblastic bone formation [39]. These results demonstrate that reactivation of bone lining cells could explain the rapid increase in osteoblast numbers on previously quiescent bone surfaces [39].

 **V.**  **Regulation of osteocyte maturation and osteocytic osteolysis**

Immature osteocytes, which are located in the osteoid or in close proximity of the bone surface, still share many morphological features with the osteoblast [40]. However, when the osteocytes becomes deeper embedded in the mineralized bone matrix, they change their morphology to become mature osteocytes [40]. This process of osteocyte maturation is triggered by matrix mineralization [40].

As discussed above, sclerostin is an inhibitor of matrix mineralization and therefore, could also be involved in the regulation of osteocyte maturation. This was demonstrated by Atkins et al., who found that, after treating human primary osteoblast cultures with sclerostin, cells increased their *E11* expression (pre-osteocyte marker), while decreasing dentin matrix acidic phosphoprotein 1 (*DMP1)* and *SOST* expression (mature osteocyte markers) [30].

In the bone, sclerostin is produced by the mature osteocytes, where its expression is regulated by a wide variety of factors, including local cytokines [41], hormones such as PTH [18] and estrogen [19], and mechanical loading [42]. Under unloading conditions, sclerostin is produced by the osteocytes in order to regulate re-shaping of the osteocytic lacunae by ‘osteocytic osteolysis’ [43]. The proteins that are upregulated by sclerostin correspond to those produced by the osteoclasts, including carbonic anhydrase 2 [43], cathepsin K [43,44], tartrate-resistant acid phosphatase [43,44] and C-terminal collagen telopeptide [31]. This process of osteocytic osteolysis has important effects on bone physiology, not only by releasing calcium from the bone matrix, which is shown to be crucial during lactation [44], but also by affecting mechano-sensation and bone turnover (reviewed by Tsourdi et al. [45]).

 **VI.**  **Stimulation of bone resorption**

Research has shown that the canonical Wnt/β-catenin signaling is a critical regulator of osteoclastogenesis [46,47,48]. During the inhibition of canonical Wnt signaling, the expression of OPG is decreased, thereby increasing the RANKL/OPG ratio, and, thus, bone resorption [49]. Wei et al. showed that, in response to RANKL, β-catenin in osteoclast precursors is downregulated, which is needed to allow the differentiation of osteoclast precursors towards mature osteoclasts [46]. Furthermore, Weivoda et al. demonstrated that osteoclast lineage cells express canonical Wnt receptors [48], indicating that sclerostin might have direct effects on osteoclast formation and maturation.

## 3. The Role of Sclerostin in Ectopic Calcification

Ectopic calcification (i.e., inappropriate mineralization, which can occur in various soft tissues) is normally prevented by the presence of local and systemic calcification inhibitors. The absence of one or more of these calcification inhibitors allows the development of calcifications that are typically composed of calcium phosphate salts, such as hydroxyapatite. Although ectopic calcifications can develop in various parts of the body, cardiovascular tissues, skin, kidney and tendons seem particularly prone [50]. The deposition of calcification in tissues, which do not calcify under normal physiological conditions, may lead to serious adverse clinical effects.

### 3.1. Vascular Calcification

Calcification of the vascular tree is associated with aging, however, is accelerated in the presence of diabetes, chronic kidney disease (CKD), osteoporosis, dyslipidemia and certain genetic diseases. In these patient populations, vascular calcification importantly contributes to increased morbidity and mortality. Vascular calcification is an actively regulated and complex process that shares many similarities with bone development and metabolism. During the vascular calcification process, vascular smooth muscle cells lose their smooth muscle cell markers (e.g., α smooth muscle actin and smooth muscle protein 22 α), and obtain the characteristics of bone-like cells—a process called osteochondrogenic transdifferentiation. This goes along with loss of mineralization inhibitors, the formation of calcifying matrix vesicles, degradation of the extracellular matrix and vascular smooth muscle cell (VSMC) death [51,52,53]. Since the canonical Wnt signaling is known to be a crucial regulator of bone turnover, this signaling pathway could therefore also be involved in vascular calcification.

Two different types of vascular calcification can be distinguished depending on their location in the vascular wall: intima calcification, which is associated with atherosclerosis, and media calcification, or Mönkeberg’s sclerosis.

Intima calcification, which frequently affects the aorta and large elastic arteries, is characterized by patchy calcifications in the vicinity of lipid/cholesterol deposits. Plaque growth, which goes along with progressing intima calcification, causes a narrowing of the arterial lumen, leading to ischemia in the downstream organs and tissues. Acute rupture of the plaque’s fibrous cap, on the other hand, can result in thrombosis or infarction. To which extent the presence of calcification influences plaque stability is still controversial (reviewed by Barrett et al. [54]). Experimental evidence demonstrated the involvement of the canonical Wnt signaling in atherosclerosis, more specifically its role in endothelial dysfunction [55,56,57,58,59], macrophage activation [60,61,62,63] and VSMC proliferation and migration [64,65]. Considering the involvement of the canonical Wnt signaling in the development and progression of atherosclerosis, a possible role for sclerostin could be reserved. Research by Leto et al. identified sclerostin expression in atherosclerostic plaques in patients with and without type 2 diabetes [66]. Furthermore, in type 2 diabetes patients, there is an association between the presence of atherosclerotic disease and serum sclerostin levels [67]. Since Krishna et al. found that sclerostin decreases the expression of genes involved in matrix degradation and calcification, and thereby inhibits atherosclerosis [68], it is likely that sclerostin could function as an inhibitor of intimal vascular calcification.

**Figure 2:**
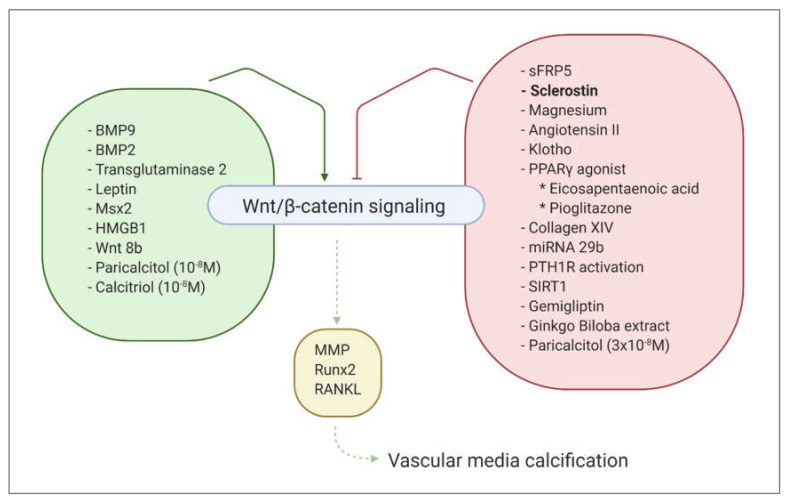
Overview of activators and inhibitors of Wnt/β-catenin signaling pathway. BMP9: Bone-Morphogenetic Protein 9, BMP2: Bone-Morphogenetic Protein 2, Msx2: Msh homeobox 2, HMGB1: High Mobility Group Box 1, sFRP5: secreted Frizzled-Related Protein 5, PPARγ: Peroxisome Proliferator-Activated Receptor γ, PTH1R: Parathyroid Hormone 1 Receptor, SIRT1: Sirtuin1, MMP: Matrix Metalloproteinase, Runx2: Runt-related transcription factor 2, RANKL: Receptor Activator of Nuclear Factor Kappa-Β Ligand.

Vascular media calcification, also named arteriosclerosis, or Mönckebergs’ sclerosis, is characterized by concentric calcifications in the tunica media of large elastic arteries, medium-sized visceral and renal arteries, as well as in small transitional arteries [69,70,71]. The principal consequences are increased wall thickness, arterial stiffening and development of left ventricular hypertrophy. This type of calcification, although frequently observed with aging in the general population, is strongly pronounced in patients with CKD, diabetes mellitus and other metabolic disorders. In CKD patients, elevated calcium and phosphate levels exert direct effects on VSMCs in order to stimulate vascular calcification. This is done by promoting osteochondrogenic transdifferentiation of VSMCs, VSMC apoptosis, matrix vesicle release, loss of calcification inhibitors and degradation of the extracellular matrix. Similar to intimal calcification, the canonical Wnt signaling pathway is also activated in vascular media calcification, as demonstrated by several studies [72,73,74,75,76,77,78,79,80,81]. Its activation is further regulated by numerous factors, as described in Figure 2. Most of the factors that activate the canonical Wnt signaling act by directly stimulating β-catenin expression and/or accumulation in the nucleus [73,78,82,83,84]. Other mechanisms, such as upregulation of Wnt ligands [77,85] and decreased expression of Wnt inhibitors [85], however, are also involved. Activation of the Wnt/β-catenin signaling, in turn, promotes vascular calcification by different mechanisms, amongst which are: (1) the modulation of Runx2 gene expression, which stimulates osteochondrogenic transdifferentiation of VSMCs [86]; (2) the induction of matrix metalloproteinases [79]; and (3) the upregulation of RANKL expression [81]. Given the important clinical consequences of vascular media calcification, many researchers have been investigating the inhibitors of the canonical Wnt signaling in the context of vascular media calcification. One of the mechanisms to inhibit activation of this signaling cascade is by preventing Wnt ligands to bind to their receptors. This can be achieved either by: (1) binding of secreted Frizzled-related proteins (sFRP) to Wnt ligands, which makes it impossible for Wnt ligands to bind to the Fz receptor [76],; or possibly (2) also by binding of sclerostin to the LRP4/5/6 receptors, which makes these receptors unavailable for Wnt-ligands [87,88]. Other mechanisms that have been described to inhibit vascular calcification by inactivating the Wnt/β-catenin signaling include magnesium [89] (and angiotensin II, which stimulates magnesium influx [90]), Klotho [91], PPARγ agonists [92,93], Collagen XIV [94], microRNAs [75,95,96], PTH receptor activation [97], Sirtuin1 [98,99], the anti-diabetic drug gemigliptin [100] and Ginkgo Biloba extract [88]. Data regarding the role of calcitriol and its analog paricalcitol in the activation of Wnt/β-catenin signaling and the vascular calcification process are conflicting. It is likely that their effects on vascular calcification development are dose-dependent [101,102].

The regulating factor that during the last years received the most attention, with regard to vascular calcification and Wnt-signaling, is sclerostin. Most research investigating the role of sclerostin in vascular media calcification is performed in the setting of CKD. In this patient population, the rapidly progressive development of vascular calcification is associated with significant morbidity and mortality. Compared to healthy individuals, end-stage renal disease (ESRD) patients have serum sclerostin levels that are three to four times higher [103,104]. This, however, does not seem to be attributed to reduced clearing by the kidneys; on the contrary, it was demonstrated that the elimination of sclerostin by the kidneys increases with declining kidney function [105]. A plausible explanation for these increased circulating sclerostin levels is an enhanced production of sclerostin. In this context, the osteocytes in the bone, which are the main producers of sclerostin, are to be considered first. One study in juvenile cystic kidney (jck) mice, a genetic model that develops progressive renal failure, showed that, in these mice, the number of osteocytes that express sclerostin increases during the very early stages of the disease compared to WT mice [106]. However, sclerostin expression returns to normal during the further course of the disease [106]. Since deterioration of the kidney function goes along with the development of vascular media calcification, transdifferentiated VSMCs could also be responsible for the observed increase in serum sclerostin levels. Although some studies did not find sclerostin to be expressed in calcified arteries [107], several others detected sclerostin mRNA and protein in calcified vessels [87,108,109]. It was suggested that ‘vascular’ sclerostin can contribute to increased serum levels in a non-CKD model for vascular calcification. The study, by De Maré et al., showed that serum sclerostin levels were increased, along with an increased expression of sclerostin in the vasculature, but without changes in the number of sclerostin-producing osteocytes [87]. Also, in calciphylaxis, a rare type of ectopic mineralization in which the calcifications are located in the medial layer of cutaneous arterioles, sclerostin is expressed in the calcified tissue and increased serum levels are observed [110,111].

Whether circulating sclerostin levels could function as a marker for the degree of vascular calcification remains unclear. Some studies find no association [112], while others find a positive [107,113,114,115,116,117,118] or negative correlation between circulating sclerostin levels and the extent of vascular calcification [119,120]. Several elements could have contributed to these conflicting results. Studies investigated the presence of vascular calcification at different anatomical locations, and used different statistical analyses (by adjusting for different potential confounding factors). In addition, differences in the time period between administration of enoxaparin (or other low molecular weight heparins that are used as anticoagulants) and blood collection could also be important, since enoxaparin stimulates the release of sclerostin into the circulation [121]. This also implies that the heterogeneity of study populations—i.e., patients at different CKD-stages, with or without dialysis treatment, whether or not they receive low molecular weight heparins—contributes to the observed inconsistency. Lastly, it is known that there are large discrepancies between sclerostin assays [122,123,124]. The antibodies that are used in the distinct assays bind different epitopes, therefore, some antibodies will capture only the intact sclerostin molecule, whilst others might also bind to sclerostin fragments.

The exact consequences of increased serum sclerostin levels in CKD patients are not unequivocally recognized. Studies investigating the association between serum sclerostin and (cardiovascular) mortality reported conflicting results, ranging from no association [118,125,126,127] to a positive one [128], as well as a negative association [119,129,130,131,132]. These inconsistent results could again be attributed to the use of different immunoassays to determine the serum sclerostin concentration. Moreover, the advanced/terminal condition of many CKD patients further complicates these kind of survival studies.

**Figure 3:**
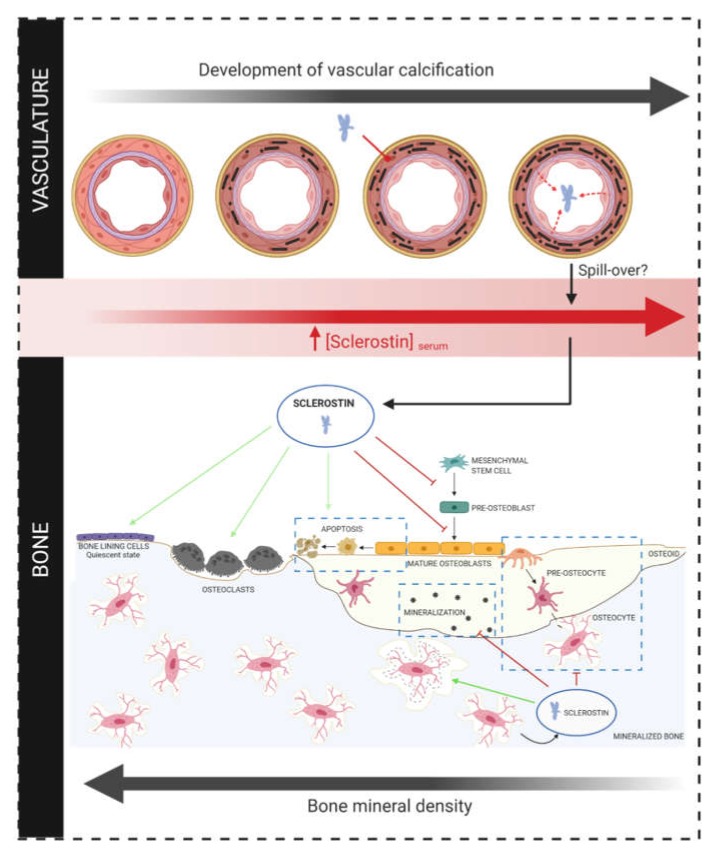
Hypothesized contribution of sclerostin in the calcification paradox. In a calcified artery, sclerostin is produced by transdifferentiated vascular smooth muscle cells [87,108]. It is hypothesized that this locally produced sclerostin can spill-over into the circulation, thereby contributing to increased serum sclerostin levels [87,134]. Via the circulation, this increased amount of sclerostin reaches the bone compartment, where it might be involved in the inhibition of bone formation/mineralization/turnover [87].

Conflicting results in the association between circulating sclerostin levels and severity of calcification/cardiovascular mortality could also be linked to sclerostin’s primary role, i.e., effects at the level of the bone. In addition to its potential effects on cardiovascular mortality, by altering the vascular calcification process, increased serum sclerostin levels can also have consequences at the level of the bone. In CKD patients, but also in osteoporosis patients, ectopic vascular calcification is frequently accompanied by a reduced bone mineral density and disturbed bone turnover. This seemingly contradictory association is called the calcification paradox [133]. During this pathological process, the regulation of several molecules and pathways involved in calcium and phosphate homeostasis (FGF23, PTH, Vit D, Klotho) is disturbed, leading to renal osteodystrophy/osteoporotic bone loss on the one hand, and ectopic calcification on the other hand. Also sclerostin might be involved in this process, since it is hypothesized that sclerostin produced in the vasculature can spill over into the circulation, via which it reaches the bone compartment, where it inhibits bone formation/mineralization/turnover [87,134] (Figure 3), thereby inhibiting the bone’s buffering capacity for calcium and phosphate, and making it available for deposition in the vessel wall.

### 3.2. Aortic Valve Calcification

Compared to patients with a normal renal function, there is an increased prevalence and accelerated progression of aortic valve calcification (AVC) in ESRD patients [135]. One study suggested the involvement of the canonical Wnt/β-catenin-signaling pathway in the differentiation of aortic valve interstitial cells into osteoblast-like cells [136]. A limited number of studies investigated the role of sclerostin in aortic valve calcification. One study demonstrated that the expression of sclerostin, which, in the aortic valve is located adjacent to the areas of calcification, was strongly associated with AVC in hemodialysis patients [137]. The same research group also compared the sclerostin expression in aortic valves, as well as serum levels in patients with and without aortic valve calcification [138]. Higher serum sclerostin levels were observed in patients with aortic valve calcification. Moreover, in these patients, sclerostin expression in the aortic valves was increased, as demonstrated by immunohistochemistry and gene expression analysis [138]. Lastly, Ji et al. demonstrated that serum sclerostin levels increase gradually, along with renal deterioration in CKD stage 3–5 patients, and that sclerostin can be an independent risk factor for aortic valve calcification in this patient population [139].

### 3.3. Genetic Disorders Characterized by Ectopic Calcification

Diffuse ectopic calcifications, located in all kinds of soft tissues, such as the arteries, myocardium, joints, liver and brain, are often the result of mutations in genes encoding for/or involved in the production of calcification inhibitors (MGP [140], pyrophosphate [141,142,143]), or proteins involved in phosphate regulation [144,145,146]. Data regarding the role of sclerostin during this ectopic calcification process are very limited. However, in a mouse model for primary familial brain calcification, it was demonstrated that sclerostin is expressed in vessel-associated calcifications in the brain [147].

### 3.4. Cancer-Associated Calcifications

Micro-calcifications are mainly observed in primary breast cancer [148], but can also be present in other types of cancer, such as prostate, thyroid and lung cancer [149,150,151]. Often, bone-related proteins are identified in these mineralized foci, indicating that this process might be similar to the physiological bone formation process [148]. To our knowledge there is currently no research investigating the role of sclerostin in this process.

### 3.5. Intracranial Calcification

Intracranial calcification refers to mineral depositions that are located within the brain parenchyma or the blood vessel wall [152,153]. This causes neuronal death and gliosis, resulting in a progressive deterioration of cognitive and motor skills, including dementia, Parkinsonism, psychosis, mood swings, dystonia and spastic paralysis [153]. Calcifications are mostly observed in the basal ganglia, but also in the thalamus, cerebellum and subcortical white matter [154]. The etiology behind this ectopic calcification process can be genetic predisposition, but also inflammatory and metabolic disorders [153]. Aberrant canonical Wnt signaling, has been shown to be involved in the pathogenesis of adamantinomatous craniopharyngiomas [155]. In these patients, gene expression analysis showed an increased expression of the canonical Wnt inhibitors, sFRP1 and Dickkopf-related protein 3 (DKK3) [156]. Further research is needed to determine the role of sclerostin in this disorder, and more generally in intracranial calcification.

## 4. Prospects

The role of sclerostin in ectopic calcification clearly needs to be investigated further, especially since an anti-sclerostin antibody, Romosozumab (EVENITY^®^), was approved last year for medical use in Japan, South Korea, Australia, Canada, US and the European Union [157]. This antibody binds to sclerostin, thereby preventing the interaction between sclerostin and LRP4/5/6 co-receptors. In osteoporosis patients, this monoclonal antibody successfully increased bone mineral density by inducing bone formation and decreasing bone resorption [24]. However, notwithstanding its clear benefits to bone health, there are concerns regarding the cardiovascular safety [158].

The ARCH study in postmenopausal women, compared weekly treatment with alendronate for 24 months versus monthly injection with Romosozumab for 12 months, followed by 12 months of alendronate treatment. Despite being superior in preventing fractures, treatment with Romosozumab resulted in a higher incidence of cardiovascular adverse events during the first year, compared to alendronate treatment [159]. These observations might point to a protective function of sclerostin in vascular calcification. As described above, studies demonstrated up-regulated expression of sclerostin in calcified vessels, and, although the function of sclerostin in the vasculature is not fully understood [87,108,109], this upregulation of sclerostin is hypothesized to be a negative feedback mechanism to prevent further calcification, which is also the case in bones [87,134]. In addition to the reduced hydroxyapatite formation by inhibiting canonical Wnt signaling, sclerostin might also prevent further calcification by stimulating dedifferentiated VSMCs to obtain adipocyte-like characteristics. Evidence for the involvement of sclerostin in adipogenesis comes from several in vitro [160,161] and in vivo studies [161,162,163]. Additionally, in the setting of ectopic vascular calcification, proteomic analysis demonstrated the upregulation of adipocyte-related proteins [87]. Amongst these proteins might be PPARγ, an important inducer of adipogenesis and known to be upregulated by sclerostin [160]. Furthermore, studies have shown that PPARγ activation is able to counteract vascular calcification [164,165].

Although preclinical studies did not detect any soft tissue mineralization due to the anti-sclerostin antibody treatment, it needs to be mentioned that radiography is not the most sensitive method to detect vascular calcification [166,167]. Additionally, in sclerosteosis and Van Buchem disease patients, no impairment of cardiac or vascular function has been reported. However, studies have shown that in order to compensate for the loss of sclerostin in sclerosteosis and Van Buchem disease patients, as well as in *Sost* KO mice, expression of other Wnt inhibitors such as Dickkopf-related protein 1 (DKK1) are upregulated [168,169]. Overexpression of DKK1 is not able to prevent excessive bone formation, however, it might be sufficient to prevent ectopic calcification. Furthermore, due to the high bone formation rate in these patients, in combination with reduced osteoclastic activity, most of the calcium and phosphate will be stored in the bone compartment, and will therefore not be available for the development of ectopic calcification. Another explanation for the (seemingly) increased incidence of cardiovascular events due to Romosozumab treatment, is the comparison with alendronate. This latter drug has potential cardioprotective effects [170], however, published results are contradictory [171,172]. Given the ambiguity regarding the cardiovascular risk when treated with Romosozumab, the prescribing information included a warning stating that “Romosozumab may increase the risk of myocardial infarction, stroke and cardiovascular death” [173]. Until the role of sclerostin in ectopic calcification has not been fully clarified, it is advised to cautiously outweigh benefits and risk in patients with cardiovascular risk factors.

## Abbreviations

SOST	gene encoding sclerostin
Wnt	Wingless-related integration site
Fz	Frizzled
LRP5/6	Low-density Lipoprotein Receptor-related Protein 5/6
TCF	T-cell factor
LEF	Lymphoid Enhancer-binding factor
PPARγ	Peroxisome Proliferator-Activated Receptor γ
C/EBPα	CCAAT/Enhancer-Binding Protein α
Runx2	Runt-related transcription factor 2
OPG	Osteoprotegerin
RANKL	Receptor Activator of Nuclear Factor Kappa-Β ligand
PTH	Parathyroid hormone
LRP4	Low-density Lipoprotein Receptor-related Protein 4
P1NP	Procollagen type 1 N-terminal Propeptide
BsAP	Bone-specific Alkaline Phosphatase
SIBLINGS	Small Integrin Binding Ligand N-Glycoproteins
MEPE	Matrix Extracellular Phosphoglycoprotein
ASARM	Acidic Serine Aspartate-Rich MEPE-associated motif
PHEX	Phosphate-regulating neutral Endopeptidase, X-linked
KO	Knockout
WT	Wild type
Bax	Bcl2- associated X protein
DMP1	Dentin matrix acidic phosphoprotein 1
CKD	Chronic kidney disease
VSMC	Vascular smooth muscle cell
sFRP	secreted Frizzled-related protein
ESRD	End-stage renal disease
Jck	Juvenile cystic kidney
FGF23	Fibroblast growth factor 23
MGP	Matrix γ-carboxyglutamic acid
AVC	Aortic valve calcification
DKK3	Dickkopf-related protein 3
DKK1	Dickkopf-related protein 1
